# Updated rapid risk assessment from ECDC on the risk related to the spread of new SARS-CoV-2 variants of concern in the EU/EEA – first update

**DOI:** 10.2807/1560-7917.ES.2021.26.3.2101211

**Published:** 2021-01-21

**Authors:** Eurosurveillance editorial team

**Affiliations:** 1 European Centre for Disease Prevention and Control (ECDC), Stockholm, Sweden

The European Centre for Disease Prevention and Control (ECDC) provides regularly updated information on coronavirus disease (COVID-19) relevant to Europe on a dedicated webpage. Besides general information including infographics, daily case counts, and maps with disease distribution, examples of latest updates comprise: Sequencing of SARS-CoV-2, Rapid assessment of laboratory practices and needs related to COVID-19, COVID-19 vaccination and prioritisation strategies in the EU/EEA. ECDC also publishes regular risk assessments and the Box below contains the summary from the first update on the risk related to the spread of new SARS-CoV-2 variants of concern in the EU/EEA published on 21 January 2021.

Box **Summary of the ECDC rapid risk assessment on the risk related to the spread of new SARS-CoV-2 variants of concern in the EU/EEA from 21 January 2021**

Viruses constantly change through mutation and variations in the SARS-CoV-2 virus, due to evolution and adaptation processes, have been observed worldwide. While most emerging mutations will not have a significant impact on the spread of the virus, some mutations or combinations of mutations may provide the virus with a selective advantage, such as increased transmissibility or the ability to evade the host immune response. In this update we report new information on the spread of three virus variants (VOC 202012/01, 501Y.V2 and variant P.1). These variants are considered to be of concern because of mutations which have led to increased transmissibility and deteriorating epidemiological situations in the areas where they have recently become established.

Based on the new information, the risk associated with the introduction and community spread of variants of concern has been increased to **high/very high** and the options for response have been adjusted to the current situation.

**Variants of concern**

VOC 202012/01 was first identified as being of concern in the south of the United Kingdom (UK) in December 2020. The first sample in which it could be identified has been traced back to September 2020. Since then, it has become the predominant variant circulating in the UK. It is characterised by a significantly increased transmissibility, which has contributed to increases in incidence, hospitalisations and pressure on the healthcare system since the second half of December 2020. The UK has implemented stricter nonpharmaceutical interventions (NPIs) to reduce transmission. Preliminary studies indicate that there is no evidence that VOC 202012/01 is associated with a significantly different infection severity or that it disproportionally affects certain age groups more than the previous circulating viruses. However, as a result of the increased incidence, by January 2021 the UK had reported the highest daily COVID-19 mortality since the start of the pandemic. Ireland, where local circulation of VOC 202012/01 has also recently been identified, has experienced an increase in case numbers and hospitalisations, growing pressure on the health system and has also had to implement stricter NPIs. Denmark has also observed community transmission of VOC 202012/01 and in response has strengthened NPIs and prolonged measures throughout January 2021.

The variant 501Y.V2 was first identified in South Africa in December 2020, where it is now the most prevalent variant. Preliminary results indicate that this variant may also have an increased transmissibility. However, as for VOC 202012/01, at this stage it is uncertain whether the 501Y.V2 variant causes a change in disease severity. As per 19 January 2021, 501Y.V2 has been identified in 10 EU/EEA countries. One cluster of this variant is currently being investigated in France. In addition to France, Israel and the UK have also reported cases or clusters of non-travel-related 501Y.V2 cases. The remaining cases identified in the EU/EEA have mostly been travel-related, but not only from South Africa.

The P.1 variant has so far only been identified in Brazil, and in travellers from Brazil (mostly from the Amazonas State) reported in Japan and South Korea. The capital of Amazonas, Manaus, is currently experiencing an upsurge in COVID-19 cases, putting significant pressure on the healthcare system.

The under-ascertainment of SARS-CoV-2 infections in general, and the very small proportion of cases undergoing sequencing in most EU/EEA countries, may lead to a large under-ascertainment of the true number of VOC 202012/01, 501Y.V2 and P.1 infections, and other potential variants that may contribute to rapid epidemiological changes.

**Risks associated with virus variants**

ECDC assesses the probability of the introduction and community spread of variants of concern in the EU/EEA as **very high** due to their increased transmissibility. Such an increased transmissibility is likely to lead to an increased number of infections. This, in turn, is likely to lead to higher hospitalisation and death rates across all age-groups, but particularly for those in older age groups or with co-morbidities. Consequently, stricter NPIs are needed to reduce transmission and relieve the pressure on healthcare systems. Therefore, the impact of introduction and community spread is considered to be **high**. The overall risk associated with the introduction and community spread of variants of concern is therefore assessed as being **high/very high**.

**Options for response**

Member States should continue to monitor local changes in transmission rates or infection severity to identify and assess the circulation and impact of variants. In order to detect introductions of known variants, as well as the emergence of new variants, Member States need to increase the level of surveillance and sequencing of a representative sample of community COVID-19 cases.

Member States should prepare laboratories for increased testing turnover. Laboratories should consider implementing diagnostic pre-screening for variants of concern (e.g. N501Y and deletion 69-70), ensure resources are available to manage an increasing number of requests for detection and characterisation of COVID-19 samples, and increase sequencing capacity by making use of all possible sequencing capacity from clinical, diagnostic, academic and commercial laboratories across different sectors.

In order to control the spread and impact of the SARS-CoV-2 emerging variants with increased transmissibility, a combination of compliance with NPIs - including potentially stricter NPIs than those currently in place – and strengthened case detection with contact tracing is required. Since the population groups driving transmission will not be targeted with vaccination for some months, Member States are recommended to be very cautious about relaxing NPIs. Furthermore, in light of the evidence of substantially higher transmissibility of the new variants of concern, national authorities should rather be ready to enforce even stricter measures, communicating and engaging with the population to encourage compliance. In general, contact tracing should be reinforced, and its scope widened in relation to cases suspected to be infected with new variants.

In order to slow down the importation and spread of the new SARS-CoV-2 variants of concern, ECDC recommends that non-essential travel should be avoided. In addition to recommendations against nonessential travel, and restrictions on travel for those infected, travel measures such as the testing and quarantining of travellers should be maintained, in particular for travellers from areas with a higher incidence of the new variants. If sequencing is inadequate to exclude the possibility of a higher incidence of the new variants, as per ECDC guidance on genomic sequencing, proportionate travel measures should also be considered from areas where there is a continued high level of community transmission.

Member States should prepare their healthcare systems for a further escalation in demand due to the increased transmissibility of the new variants of concern.

Member States are encouraged to accelerate the pace of vaccination for high-risk groups, such as the elderly and healthcare workers. At this stage, vaccination should be focused on protecting those most at risk from severe disease, and reducing morbidity, mortality and the burden on healthcare systems. It is important to use the available vaccines to provide protection for those who are most vulnerable and for key workers against the current circulating virus variants in the EU/EEA, and hopefully also against one or all of the new variants of concern. Assessment of VOC 202012/01 suggests cross-immunity is present, while investigations into the other variants of concern are still on-going. Member States should monitor vaccine effectiveness for these new variants. Breakthrough infections should be monitored, carefully investigated (including sequencing the virus variant causing breakthrough infection), and reported to public health and regulatory agencies to allow for an overview at country and EU-level. In addition, Member States should explore options for optimal use of the limited number of vaccine doses.

Source: European Centre for Disease Prevention and Control (ECDC). Risk related to the spread of new SARS-CoV-2 variants of concern in the EU/EEA – first update. Stockholm: ECDC; 21 Jan 2021. Available from: https://www.ecdc.europa.eu/sites/default/files/documents/COVID-19-risk-related-to-spread-of-new-SARS-CoV-2-variants-EU-EEA-first-update.pdf
COVID-19: coronavirus disease; ECDC: European Centre for Disease Prevention and Control; EU/EEA: European Union/European Economic Area; SARS-CoV: severe acute respiratory syndrome coronavirus; UK: United Kingdom.

